# Development of a CRISPR-based cytosine base editor for restriction-modification system inactivation to enhance transformation efficiency in *Vibrio* Sp. dhg

**DOI:** 10.1186/s13036-025-00500-4

**Published:** 2025-04-09

**Authors:** Yang Jun Shon, Dongyeop Baek, Su Bin Jin, Woo Jae Kim, Gyoo Yeol Jung, Hyun Gyu Lim

**Affiliations:** 1 Department of Chemical Engineering, Pohang University of Science and Technology, 77 Cheongam-Ro, Nam-Gu, Pohang, Gyeongbuk 37673 Korea; 2 School of Interdisciplinary Bioscience and Bioengineering, Pohang University of Science and Technology, 77 Cheongam-Ro, Nam-Gu, Pohang, Gyeongbuk 37673 Korea; 3 Department of Biological Sciences and Bioengineering, Inha University, 100 Inha-Ro, Michuhol-Gu, Incheon, 22212 Korea

**Keywords:** CRISPR, Base editor, *Vibrio*, Multiplex editing, Restriction-modification, Transformation efficiency

## Abstract

**Background:**

*Vibrio* sp. dhg is a fast-growing, alginate-utilizing, marine bacterium being developed as a platform host for macroalgae biorefinery. To maximize its potential in the production of various value-added products, there is a need to expand genetic engineering tools for versatile editing.

**Results:**

The CRISPR-based cytosine base editing (CBE) system was established in *Vibrio* sp. dhg, enabling C: G-to-T: A point mutations in multiple genomic loci. This CBE system displayed high editing efficiencies for single and multiple targets, reaching up to 100%. The CBE system efficiently introduced premature stop codons, inactivating seven genes encoding putative restriction enzymes of the restriction-modification system in two rounds. A resulting engineered strain displayed significantly enhanced transformation efficiency by up to 55.5-fold.

**Conclusions:**

Developing a highly efficient CBE system and improving transformation efficiency enable versatile genetic manipulation of *Vibrio* sp. dhg for diverse engineering in brown macroalgae bioconversion.

**Supplementary Information:**

The online version contains supplementary material available at 10.1186/s13036-025-00500-4.

## Background

*Vibrio* sp. dhg, a marine bacterial isolate, has gained much attention as a platform host for macroalgae biorefineries [1]. It grows much faster than model hosts (e.g., *Escherichia coli*) with higher specific sugar uptake rates (up to 2-fold) and tolerance to high salinity (up to 100 g L^− 1^ NaCl) [1]. In addition to these characteristics, it can rapidly catabolize alginate, which is abundant in brown macroalgae but cannot be metabolized by conventional microbial hosts. These characteristics highlight the versatile potential of *Vibrio* sp. dhg as a novel microbial host for bioproduction utilizing brown macroalgae as substrate.

Indeed, *Vibrio* sp. dhg has been synthetically engineered by developing optimized transformation, recombineering, and predictive gene expression [1]. These efforts allowed the generation of efficient strains capable of producing diverse biochemicals. These compounds include 2,3-butanediol, lycopene [1], indole-3-acetic acid [2], itaconate [3], and citramalate [4]. However, more advanced genetic engineering tools are required, particularly focusing on multiplex genome editing, for its wide adaptation as a platform chassis.

Recently, CRISPR-guided base editing systems have been developed as a precise and efficient alternative for genome engineering in various microbes [5–7]. Base editors utilize nuclease-inactivated versions of Cas9 fused with a deaminase, enabling targeted point mutations without the need for donor DNA templates [8, 9]. In particular, with cytosine base editor, C:G-to-T:A transition mutations can be introduced at desired locus target sites. This cytosine base editor has been successfully deployed across numerous microbial strains for versatile purposes in gene inactivation [6, 10, 11], protein engineering [7, 12, 13], and expression modulation [14, 15]. Displaying high editing efficiencies, many toolkits have been established in a broad range of hosts such as *E. coli* [5], *B. subtilis* [11], *C. glutamicum* [6], *C. ljungdahlii* [16], *Streptomyces* [17], *Pseudomonas* [10], and *Roseobactor* species [18].

In this study, an efficient cytosine base editor (CBE) system was established for *Vibrio* sp. dhg as a novel genome engineering toolkit and applied to improve its transformation efficiency. This toolkit was developed using an expression system amenable to the strain and its editing function was validated on single targets, displaying high editing efficiency. The developed system was then utilized to perform multiplex editing for gene inactivation, specifically targeting the restriction-modification (RM) system by disrupting the genes encoding restriction enzymes (REases). This approach aimed to improve transformation efficiency, an important factor in the genetic manipulation of *Vibrio* sp. dhg. The developed CBE system and the generated platform strain with improved transformation efficiency are expected to enable wide adaptation of *Vibrio* sp. dhg as a microbial host for many biotechnological applications.

## Materials and methods

### Bacterial strains and culture conditions

The bacterial strains used in this study are listed in Table S1 of Additional File 1. *E. coli* Mach1 T1^R^ was used for cloning and was cultivated aerobically at 37^o^C in Luria-Bertani (LB) broth. *Vibrio* sp. dhg strains were cultivated in the LB3 (LB supplemented with additional 20 g/L NaCl) medium at 30^o^C, unless mentioned otherwise. Solid agar media additionally contain 15 g/L agar. In the use of antibiotics, chloramphenicol (Cm, 34 µg/mL for *E. coli* and 10 µg/mL for *Vibrio* sp. dhg) or/and kanamycin (Kan, 50 µg/mL for *E. coli* and 200 µg/mL for *Vibrio* sp. dhg) were supplemented for selective growth of plasmid-containing strains. For induction of the P_BAD_ promoter system, 4 g/L arabinose was added to the medium.

### Plasmid construction

Plasmids and primers used in this study are listed in Table S2 and Table S3 of Additional File 1, respectively. To express dCas9-CDA-UGIL (L at the end indicates the LVA degradation tag), the pCBE2 plasmid was constructed by inserting the fusion protein into the pACYCDuet™-1 vector harboring the P_BAD_ promoter system via Gibson assembly. The pCBE1 plasmid was constructed to express dCas9-CDA by blunt end ligation of the truncated PCR fragment without UGI. The initial single target sgRNA expression cassette targeting the super folding green fluorescent protein (sfGFP) was synthesized via Integrated DNA Technologies and inserted into the pRO1600/ColE1 vector to construct psgRNA_sfGFP by Golden Gate assembly using BsmBI digestion-ligation. The 20 nt spacer region for single targets on *rpoB* and *pyrF* was exchanged by designing respective overhangs on each end of DNA for Gibson assembly.

To construct multiplex editing sgRNA plasmids, the sfGFP dropout system [19] for spacer exchange and the modular Golden Gate cloning method [20] for multiple sgRNA cassette assembly were used. The sfGFP dropout system was constructed by inserting the sfGFP expression cassette with internal BsaI recognition sites at each end into the spacer region of sgRNA by Golden Gate assembly. SfGFP dropout to exchange for the desired target spacer was performed by spacer-containing oligonucleotide pair annealing, BsaI digestion of the sfGFP dropout containing sgRNA vector, and ligation of the annealed spacer into the sgRNA expression cassette. For annealing of the oligonucleotide pairs with complementary spacer sequences, 15 µL of each forward and reverse 10 µM oligonucleotide with 5 µL T4DNA ligase buffer were mixed in a total 50 µL-reaction and annealed by incubation to 95^o^C for 15 min then slowly cooled to room temperature (~ 1 h). The sfGFP dropout cassette was restricted by BsaI digestion followed by purification of the vector fragment, which was then used to ligate the annealed spacer oligonucleotide pair with the corresponding sticky ends. Ligation was conducted with 100–200 ng vector and 4–8 µL of annealed oligonucleotide insert in a 20 µL T4 DNA ligation reaction. gRNAs are listed in Table S4 and sequences for premature stop codon formation were designed using “BE-Designer” of the CRISPR RGEN Tools [21]. For modular Golden Gate cloning, the Level 0 single sgRNA modules and Level 1 multiplex sgRNA destination vectors were constructed with corresponding sticky ends listed for modular assembly by BsmBI digestion-ligation. Then, the Level 0 sgRNA expression cassettes were assembled into each Level 1 destination vector according to the appropriate sticky ends by BsmBI modular Golden Gate cloning.

### Transformation of *Vibrio* strains via electroporation

All electroporation procedures followed a modified version of a previously established protocol [22]. A single colony was isolated and cultivated overnight at 30 °C in a 3 mL medium. The following day, saturated cells were diluted 1:100 in 5 mL fresh medium and incubated at 30 °C with shaking at 200 rpm until the OD_600_ reached 0.4–0.6 (approx. 1 h). Cell cultures were then placed on ice for 20 min. Cell pellets were collected by centrifugation at 4,830×g for 20 min at 4 °C and removal of supernatant. The cell pellets were washed three times by adding 1 mL of the electroporation buffer (680 mM sucrose, 7 mM potassium phosphate, pH 7), resuspending the cell pellets, centrifuging to collect cells, and removing the supernatant each time. After the final wash, the cells were resuspended in an electroporation buffer to reach an OD_600_ of 16. For electroporation, 90 µL of resuspended cells were aliquoted, and up to 1 µg plasmid was added to make a 100 µL mixture. This mixture was transferred into an electroporation cuvette and electroporated at 0.9 kV resulting in a time constant of 2.2–2.4 ms using a Micropulser Electroporator (Bio-Rad Laboratories). After electroporation, 1 mL prewarmed LBv2 (LB supplemented with 204 mM NaCl, 4.2 mM KCl, and 23.14 mM MgCl_2_) was added and the cells were cultivated at 37 °C for 1 h. Finally, the cells were spread on antibiotic-containing agar plates (with serial dilutions, if necessary) and incubated overnight at 37 °C.

### Determination of base editing efficiency

To perform base editing, either pCBE1 or pCBE2 plasmid and a corresponding psgRNA plasmid were serially transformed into the VDHG100 (*Vibrio* sp. dhg Δ*dns*) strain [1] by electroporation. Cells with both plasmids were grown in the presence of Cm and Kan to maintain the pCBE and psgRNA plasmids, respectively. Cells were first streaked onto solid plates and incubated at 30^o^C overnight for single colony isolation. Single colonies were picked for overnight culture in 3 mL liquid medium at 30^o^C and 200 rpm. Saturated cells were transferred by 1:100 dilution into 5 mL of the fresh medium with arabinose for induction of dCas9-CDAL or dCas9-CDA-UGIL and cultivated for 4 h. For multiplex editing, additional passaging was conducted by repeating the previous cultivation step using the induced cell culture. Single colonies of edited cells were isolated by spreading with serial dilution, or streaking onto a non-selective solid medium. To show a gain of rifampicin resistance in *rpoB* mutant strains, 50 µg/mL rifampicin was included in the solid medium. When targeting *pyrF*, 20 µg/mL uracil was supplemented in media from the induction cultivation step to single colony isolation and 1 mg/mL 5-FOA was additionally supplemented to show an increase in resistance compared to the wildtype *Vibrio* sp. dhg strain. Base editing efficiencies were measured by performing Sanger sequencing of randomly picked colonies from non-selective agar plates (Cosmogenetech, Korea).

### Determination of transformation efficiency

For the strain transformation efficiency assay, the pCBE and psgRNA plasmids were first cured via one or two rounds of serial passaging. Cells were grown to saturation at 37 °C with shaking at 200 rpm in liquid medium without antibiotics. After single colony isolation by streaking on nonselective solid medium, plasmid-free cells were selected by spotting single colonies on solid medium with and without antibiotics. In performing transformation, saturated cultures of plasmid-free cells were then diluted 1:100 in 30 mL fresh medium and cultivated in flasks until the OD_600_ reached 0.4–0.6. The cell pellets were washed three times with 10 mL of electroporation buffer under the same conditions. After adjusting the OD_600_ of the final resuspended cells to 16, 90 µL cells from each strain were aliquoted into three separate 1.5 mL microcentrifuge tubes for biologically independent replicates, and 1 µg pACYCDuet™-1 plasmid was added for electroporation. After recovery and serial dilution plating on selective media, colony-forming units (CFUs) were counted and adjusted based on the dilution factor to determine transformation efficiency.

### Genomic analysis for identifying genes which can be inactivated by base editing

Genomic DNA sequences were retrieved from the National Center for Biotechnology Information (NCBI) database (accession numbers: CP028943, CP028944, and CP028945) [23] and annotated using the Rapid Annotations of Subsystem Technology (RAST) [24]. Protein-encoding genes were selectively filtered from the annotated dataset for subsequent analyses. CBE system enables the conversion of cytosine (C) to thymine (T), which was utilized to introduce premature stop codons (TAA, TAG, and TGA) in coding sequences. On the coding strand, CAA, CAG, and CGA were targeted for conversion to stop codons. On the non-coding strand, CCA was targeted for conversion to TCA, CTA, and TTA, which corresponds to the introduction of TGA, TAG, and TAA on the coding strand. To identify potential single-guide RNAs (sgRNAs) for genome-wide inactivation, the target sequences CAA, CAG, CGA and CCA were screened within the − 20 to − 16 positions upstream the PAM sequence, corresponding to the editing window of CBEs using a 20-nucleotide spacer. The sequences and relative positions of all targetable sgRNAs for each coding sequence were identified. NGG and NGN PAM requirements were applied for CBEs using the canonical SpCas9 and PAM-flexible SpG variant, respectively. Target sequences for CBE using SpRY, a near-PAMless variant, were also analyzed without a PAM requirement. A Python script was used to perform sgRNA identification and data analysis.

## Results and discussion

### Design of the cytosine base editing (CBE) system for *Vibrio* sp. dhg

A target-specific CBE system was developed for *Vibrio* sp. dhg by utilizing cytidine deaminase fused with nuclease-inactivated Cas9 (dCas9) and uracil glycosylase inhibitor (UGI). dCas9 was chosen because it is known to be less toxic in not inducing any strand breaks compared to Cas9 or Cas9 nickases (nCas9) [5, 25, 26]; the expression of an intact Cas9 negatively affected cellular viability and was utilized to kill *Vibrio* species [27, 28]. On the other hand, *Pm*CDA1 from *Petromyzon marinus* was utilized given it shows higher catalytic activity in changing cytosine to uracil and less dependence on sequence context than other cytidine deaminases [9, 29–31]. Additionally, as an auxiliary component, uracil glycosylase inhibitor (UGI) that prevents the repair of uracil in DNA [32] was interrogated for its role in enhancing editing efficiency [5, 33]. These proteins were fused by the SH3 and 3x FLAG tag linkers into the form of a dCas9-CDAL (CBE1) fusion protein, or with an additional UGI as dCas9-CDA-UGIL (CBE2); with the LVA degradation tag attached at the C terminus of each fusion protein.

**Fig. 1:**
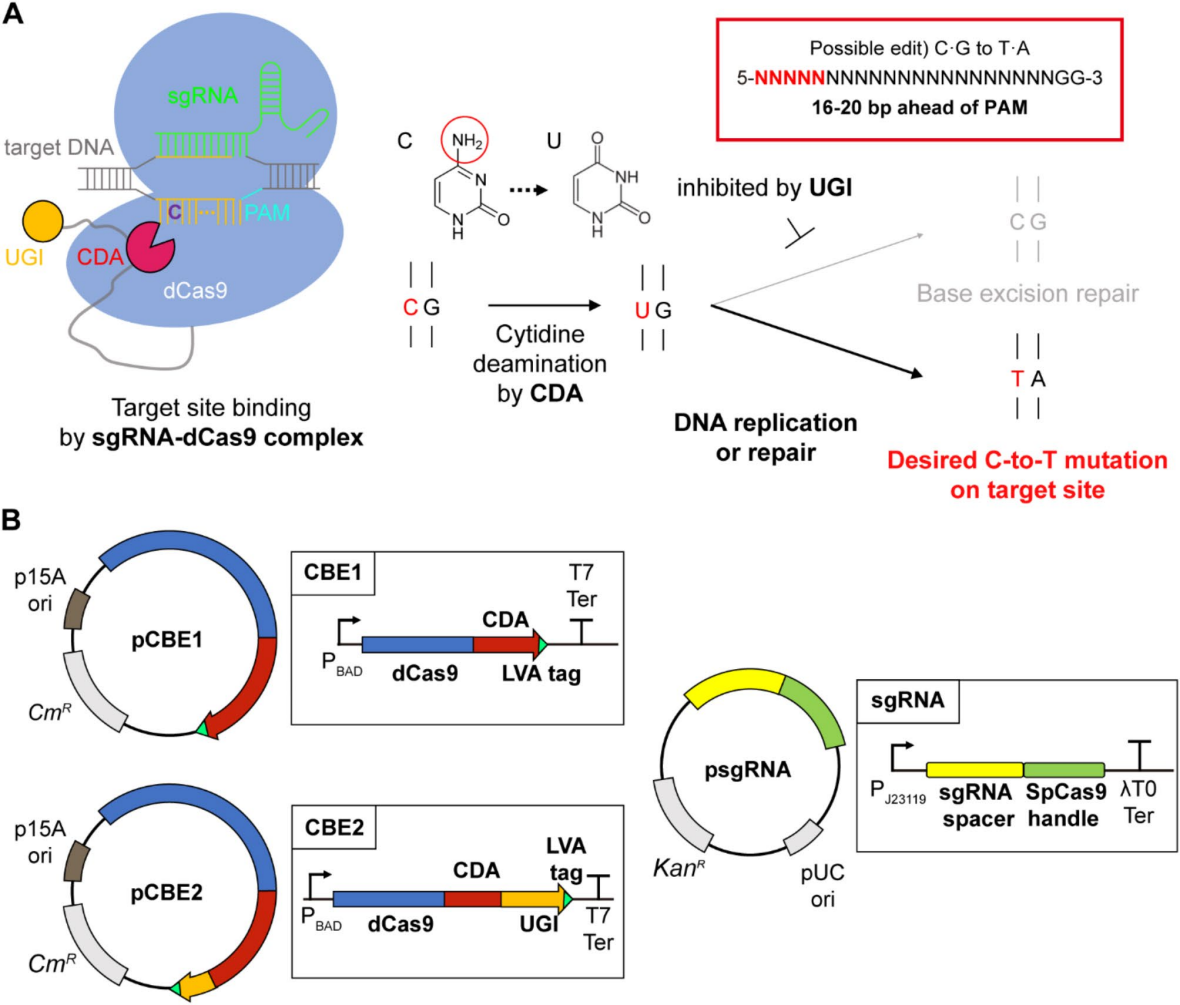
CBE system in *Vibrio* sp. dhg (**A**) Overview of the CBE mechanism, comprising the coordinated actions of dCas9/sgRNA, CDA, and UGI in C-to-T base conversion. (**B**) Schematic diagrams of the plasmids for the expression of dCas9-CDAL (pCBE1) or dCas9-CDA-UGIL (pCBE2) and sgRNA (psgRNA series)

The developed system can achieve target-specific base editing by changing the 20-nt spacer sequence of sgRNA (Fig. 1A). The sgRNA-dCas9 complex binds to the complementary site of interest. Then, CDA linked to dCas9 edits the target base by deamination of cytosine to uracil. In enhancing editing efficiency, UGI additionally linked to CDA prevents the removal of the non-DNA base, uracil, by base excision repair. This directs the eventual base change to thymine in the replication process where DNA polymerase perceives uracil as thymine. Following this mechanism, the CBE system induces C-to-T, or G-to-A targeting the antisense strand, transitions in an editing window situated 16–20 bp upstream of an NGG protospacer adjacent motif (PAM) site required for CRISPR/Cas9-based systems.

In introducing the base editing toolkit in *Vibrio* sp. dhg, a dual-plasmid expression system composed of the main editing plasmid for expressing the CBE fusion protein and the helper plasmid for expressing sgRNA, was constructed (Fig. 1B). The CBE1 or CBE2 protein was expressed in a low-copy plasmid under an arabinose-inducible promoter with an LVA degradation tag at the C-terminal, ensuring tight regulation [34] and shortened half-life [35, 36] to reduce toxicity and off-target effects. In contrast, sgRNA was expressed in a high-copy plasmid under a strong constitutive promoter. For flexibility in targeting, the crRNA spacer region of the sgRNA was designed to be easily replaceable in the helper plasmid, enabling rapid customization for different editing purposes. This dual-plasmid system also facilitates multiplex editing by allowing the expression of multiple sgRNAs from the sgRNA plasmid, thereby enhancing the versatility of the toolkit.

### Functional validation of CRISPR-based CBE system in *Vibrio* sp. dhg

**Fig. 2:**
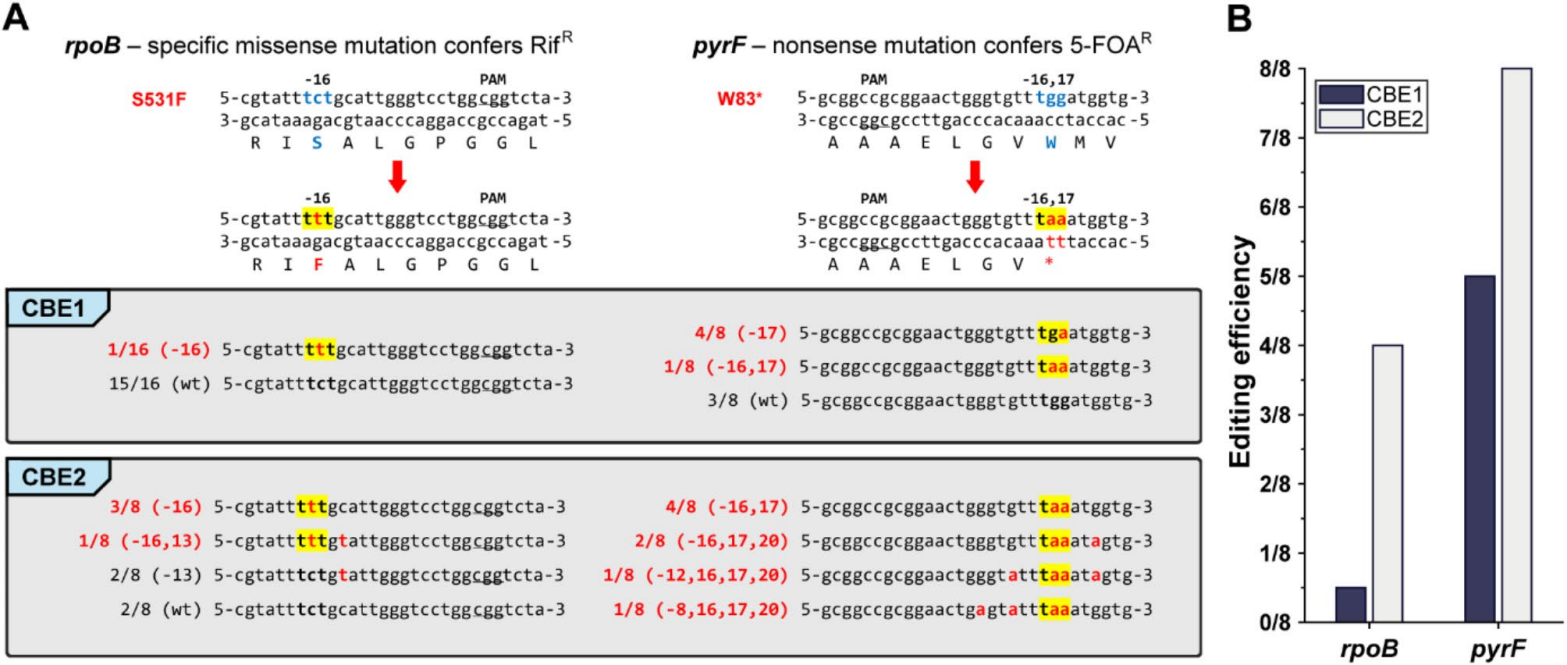
Single target base editing efficiency characterization. (**A**) Sequence outcome of single target base editing targeting *rpoB* and *pyrF* using the CBE1(without UGI) and CBE2(with UGI) system. Eight (or sixteen for the CBE1 system, *rpoB* target) random colonies were selected and analyzed to calculate editing efficiency as the ratio of colonies containing targeted mutagenesis. (**B**) Comparison of single target base editing efficiency between CBE1 (without UGI) and CBE2 (with UGI)

To initially validate functional mutagenesis of the CBE system in *Vibrio* sp. dhg, selectable genes in its genome were chosen as targets where their specific mutations are known to confer resistance to corresponding selectable agents or antibiotics. Two genes, *rpoB* and *pyrF*, were found to have a codon changeable to a stop codon within the editing window from the PAM sequence (Fig. 2A). The former encodes the RNA polymerase β subunit [37, 38]. A missense mutation at the 531st amino acid, a well-characterized site for rifampicin resistance, could be introduced using the CBE system. The latter encodes orotidine-5’-phosphate decarboxylase and was selected for its potential to confer resistance to 5-fluoroorotic acid (5-FOA) via a CBE-induced nonsense mutation at the 83rd amino acid [39, 40].

The editing efficiency of the CBE1 (dCas9-CDA) and CBE2 (dCas9-CDA-UGI) systems was assessed by targeting *rpoB* and *pyrF* (Fig. 2). Editing efficiency was calculated by sequencing randomly selected colonies and determining the proportion that carried the desired mutations: the missense mutation S531F in *rpoB* and the nonsense mutation W83* in *pyrF*. Using the CBE1 system, which includes only the core elements for base editing, the targeted mutation S531F in *rpoB* was detected in only 1 out of 16 colonies (6.25%). In *pyrF*, the targeted mutation W83* was detected in 5 out of 8 colonies (62.5%). With the inclusion of UGI in the CBE2 system, editing efficiency significantly improved, with the targeted S531F mutation present in 4 out of 8 colonies (50%) for *rpoB* and the W83* mutation in 8 out of 8 colonies (100%) for *pyrF*. These results demonstrate that UGI is an essential component for achieving high-efficiency base editing, with the CBE2 system reaching up to 100% efficiency. Consequently, the CBE2 system was selected for subsequent experiments. Consistent with the previous report [37, 38], the *rpoB*-edited strains gained resistance to rifampicin (Additional File 1: Figure S1). Similarly, *pyrF*-edited strains also showed higher resistance to 5-FOA compared to the wildtype strain in concordance with previous studies [39, 40] (Additional File 1: Figure S2). Therefore, the developed CBE system successfully introduced premature stop codons in the targeted region of the genome of *Vibrio* sp. dhg for desired purposes.

### Multiplex base editing for gene inactivation of reases in the RM system

**Fig. 3:**
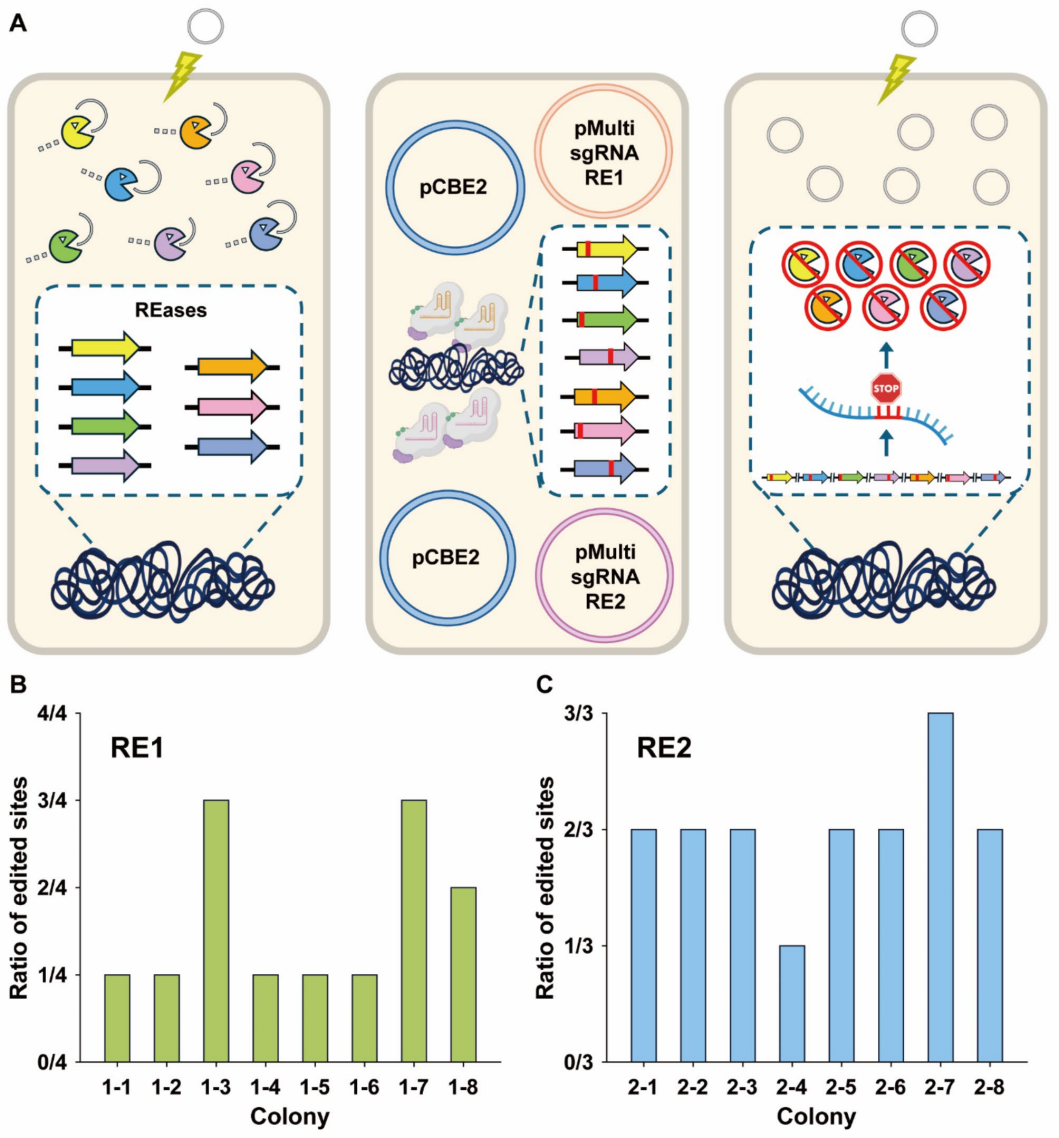
Multiplex base editing for simultaneous inactivation of REases in the RM system. (**A**) Schematic of inactivating the REases by premature stop codon formation via multiplex base editing to improve transformation efficiency - Disintegration of foreign DNA by the presence of native REases (left), Inactivation of multiple REases by the CBE system (middle), Enhancement of transformation efficiency due to the absence of active REases (right). (**B**) Multiplex base editing efficiency of RE1 targets in terms of the ratio of edited genes in each colony (green). (**C**) Multiplex base editing efficiency of RE2 targets in terms of the ratio of edited genes in each colony (blue)

In achieving high editing efficiency for single targets, the CBE system was applied for multiplex genome editing. Simultaneous targeting of multiple genes enables rapid engineering for desired genetic reprogramming. As a multiplex editing target, restriction enzymes (REases) composing the RM system were selected (Fig. 3A) given its inactivation improved transformation efficiency in bacteria such as *Caldicellulosiruptor* [41] and *Clostridium* species [42]. Bacterial RM systems recognize self-DNA by methylating at specific recognition sites by methyltransferases (MTases). If foreign DNA lacking methylation is present in cells, REases remove it to protect the host genome [43, 44]. This protection mechanism is known to lower genetic engineering efficiency during plasmid transformation or recombination using heterologous DNA fragments. Therefore, the inactivation of the system in *Vibrio* sp. dhg was warranted to improve genetic engineering efficiencies.

To deactivate these REases in *Vibrio* sp. dhg, the CBE system was utilized to introduce premature stop codons. Initially, genomic sequence analysis was performed to identify REases using the REBASE [45], NCBI [23], eggNOG [46], and RAST [24] databases. Resultantly, a total of seven REases were found: multiple *hsdR* type I restriction enzyme subunits R (DBX26_01795, DBX26_11820, DBX26_11465), *mrr* restriction endonuclease (DBX26_01820), BsuBI/PstI RE restriction endonuclease (DBX26_25330), restriction endonuclease (DBX26_05280), type III restriction-modification endonuclease (DBX26_25295) (Table 1). For their disruption, multiple sgRNAs were designed to target regions where premature stop codons can be introduced by changing C to T within the editing windows. For multiplex inactivation of REases in the RM system, targets were divided on the basis of whether they were predicted by REBASE with four targets as “RE1” and only by other databases with three targets as “RE2”. Accordingly, four and three sgRNA expression cassettes were inserted in the “pMultigRNA_RE1” and “pMultigRNA_RE2” plasmid, respectively, using the sfGFP dropout system [19] and the modular Golden Gate cloning method [20] (see Methods). The sgRNAs were all monocistronically expressed under the P_J23119_ promoter for strong expression.

Multiplex base editing was performed and its efficiency was evaluated with the two plasmids targeting the four and three genes, separately. The editing efficiency was assessed with eight random colonies by analyzing the number of edited sites per colony and the editing frequency of each target site. With the pMultigRNA_RE1 plasmid targeting four genes (Table 1), up to three simultaneous inactivation edits were observed in two of eight colonies (RE1-3. RE1-7), while the majority exhibited editing in only one gene of the targets (Fig. 3B). With the pMultigRNA_RE2 plasmid targeting three sites, most colonies had two edited sites, and one colony contained desired edits in all three targets (RE2-7) to obtain the RE2 strain (Fig. 3C). Another round of base editing was also performed to introduce a stop codon in the remaining locus in the RE1-7 strain to achieve the inactivation of all four targets in acquiring the RE1 strain.

**Table 1:** REase targets for gene inactivation in *Vibrio* sp. dhg

	Target label	Locus	Name^a, b^	Predicted source	Stop codon
RE1	RE0-1	DBX26_01795	*hsdR* type I restriction enzyme R subunit^a^	REBASE, eggNOG, RAST	W139*
	RE0-2	DBX26_01820	*mrr* restriction endonuclease^a^	REBASE, eggNOG, RAST	W104*
	RE0-3	DBX26_11820	*hsdR* type I restriction enzyme R subunit^a^	REBASE, eggNOG, RAST	R21*
	RE0-4	DBX26_25330	BsuBI, PstI restriction endonuclease^a^	REBASE, NCBI, eggNOG, RAST	W259*
RE2	RE0-5	DBX26_05280	restriction endonuclease^a^	NCBI, eggNOG, RAST	Q146*
	RE0-6	DBX26_11465	*hsdR* type I restriction enzyme R subunit^a^	eggNOG, RAST	Q62*
	RE0-7	DBX26_25295	type III restriction-modification endonuclease^b^	NCBI	Q153*

^a^Name was given according to eggNOG, RAST database annotation

^b^Name was given according to NCBI database annotation

To generate the RE3 strain, which includes the inactivation of all 7 REase targets, the pMultisgRNA_RE2 plasmid was introduced into the RE1 strain harboring pCBE2. Components were expressed to edit the remaining RE2 sites, achieving complete inactivation across all 7 targets through two separate rounds of multiplex editing using pMultisgRNA_RE1 and pMultisgRNA_RE2 (Fig. 3B). While the CBE system demonstrated high multiplex editing efficiency, variations in efficiency were observed across individual sites. For example, RE0-2 (DBX26_01820) and RE0-7 (DBX26_25295) showed high efficiency, with editing efficiency of 87.5–100%, respectively, whereas RE0-3 (DBX26_11820) and RE0-6 (DBX26_11465) exhibited low efficiency, with editing efficiency of 0–12%, respectively (Additional File 1: Figure S3). These discrepancies may arise from factors such as sequence-based gRNA affinity, genomic locus, or the differences in sgRNA expression levels within multiplex cassettes. Further investigation is needed to elucidate the underlying causes of site-specific variability and optimize multiplex editing performance.

### Inactivation of reases of the RM system on transformation efficiency

**Fig. 4:**
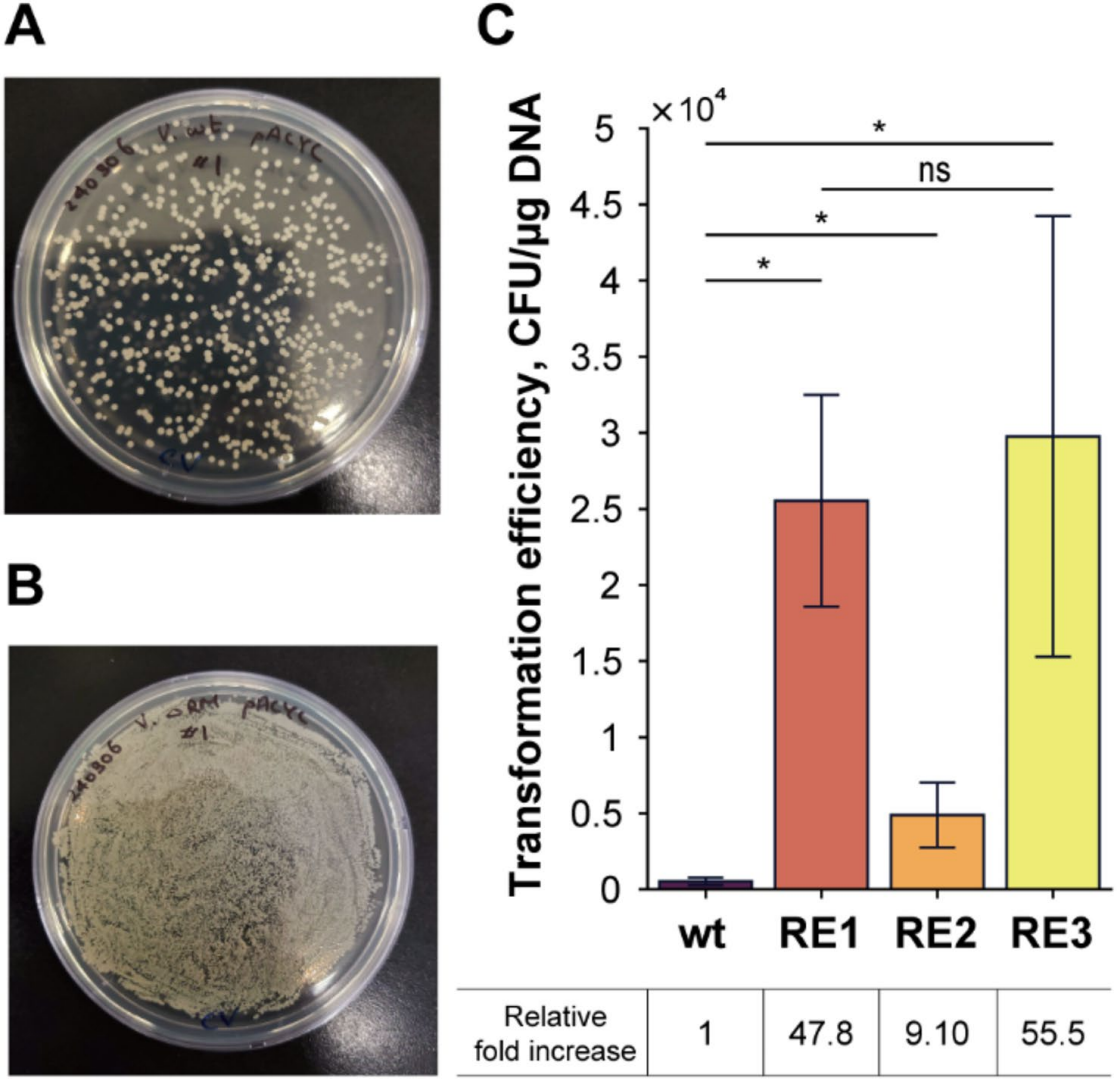
REase inactivation on transformation efficiency. Selective plate result showing the transformation of the pACYCDuet™-1 empty plasmid into (**A**) wt and (**B**) RE3. (**C**) Transformation efficiency of wt, RE1, RE2, RE3 shown as the total number of colony forming units (CFU) per µg plasmid as CFU/µg. Data results were calculated as the mean of three independent biological replicates (*n* = 3) with error bars as ± standard deviation. Statistical significance was calculated based on two-tailed Student’s t-test (*p ≤ 0.05). Table below shows the relative fold increase in transformation efficiency compared to the wt ($\:\frac{CFU/\mu g\:of\:strain}{CFU/\mu g\:of\:wt}$)

The effect of the inactivation of the REases in plasmid transformation was investigated by using the RE1, RE2, and RE3 strains. The base editing plasmids were removed by serial cultures and the pACYCDuet™-1 plasmid was introduced. As a result, all the strains displayed significant increases in transformation efficiency compared to the wildtype (Fig. 4). Notably, REase inactivation in the RE1 strain, as predicted by REBASE, resulted in a 47.6-fold increase in transformation efficiency, whereas RE2 achieved a 9.1-fold increase (Fig. 4C). However, the combined inactivation in RE3 demonstrated only a slightly higher 55.5-fold increase, indicating that RE2 targets were unable to contribute a synergistic effect. This may result from the resolution of the critical transformation barrier caused by REase activity in the RM system, particularly involving key restriction enzymes identified through REBASE predictions.

### Genomic analysis to identify gene inactivation target by base editing

**Fig. 5:**
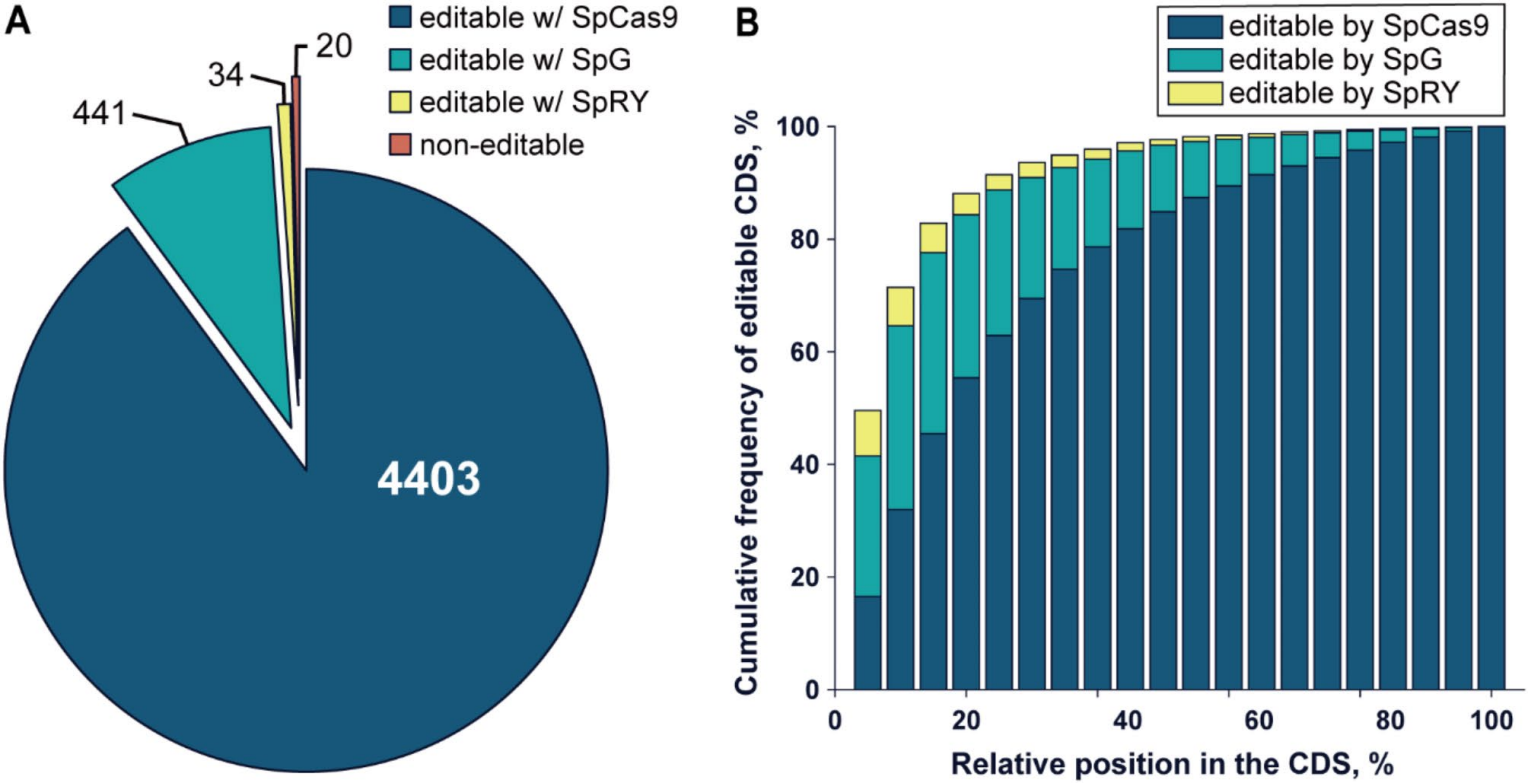
Genome-wide coverage of CBE system with Cas9 variants for gene inactivation. (**A**) Statistical coverage of genes in the *Vibrio* sp. dhg genome containing at least one targetable codon for gene inactivation. (**B**) Cumulation of editable genes by relative position in the CDS

To demonstrate the capability of the CBE system for versatile genome editing, the tool was analyzed for coverage of targetable genes in the genome for gene inactivation (Fig. 5). Base editing can be utilized for gene inactivation by premature stop codon formation in converting CGA, CAG, CAA codons in the sense strand or CCA in the antisense strand to TGA, TAG, TAA stop codons within the coding sequence (CDS) of genes. Bioinformatics analysis confirms the utility of the CBE system as a gene inactivation tool in *Vibrio* sp. dhg as 4403 (89.89%) genes out of a total of 4898 genes in the genome are targetable in forming a premature stop codon (Fig. 5A), with 84.87% of these genes capable of creating a stop codon within the first 50% of the CDS (Fig. 5B), a threshold sufficient for effective gene inactivation [10]. To further expand the coverage of editable genes, PAM-flexible or near-PAMless SpG and SpRY variants of Cas9 with NGN and NRN or NYN PAMs, respectively, can be utilized [47, 48]. Their usage enables near whole genome-wide coverage of 98.90-99.59% editable genes (Fig. 5A), having stop codons in 96.71–97.70% within the first 50% of the CDS (Fig. 5B).

Despite the high performance of the CBE system, its application is limited by the PAM site constraints inherent to CRISPR-based systems. Achieving sequence-wide coverage will require the exploitation and development of PAM-less dCas9-guided base editors and complementary nucleotide-converting systems, such as adenine base editors (ABEs), to expand the versatility of point mutagenesis applications. The successful development of a highly efficient CBE toolkit, combined with the creation of a highly electro-competent strain through REase inactivation, significantly enhanced the genetic tractability of *Vibrio* sp. dhg. These advancements enable broader utilization of this strain as a robust platform for the bioconversion of macroalgae-derived biomass into high-value products, unlocking new opportunities for sustainable bioproduction.

## Conclusions

In this study, a CRISPR-based cytosine base editing (CBE) system for *Vibrio sp. dhg* was established, enabling efficient and precise C:G-to-T:A point mutations. This system facilitated both single and multiplex gene inactivation, particularly targeting restriction enzymes (REases) of the Restriction-Modification (RM) system, which significantly improved transformation efficiency by up to 55.5-fold. Genomic analysis confirmed that 89.89% of genes in *Vibrio* sp. dhg could be targeted for inactivation using this approach, with additional targets through PAM-flexible Cas9 variants. The development of this base editing toolkit is expected to enhance the genetic tractability of *Vibrio* sp. dhg and accelerate its application as a platform strain for macroalgae biorefineries.

## Electronic supplementary material

Below is the link to the electronic supplementary material.

Supplementary Material 1

## Data Availability

No datasets were generated or analysed during the current study.

## Abbreviations

CRISPR: Clustered regularly interspaced short palindromic repeats
Cas9: CRISPR-associated protein 9
dCas9: Dead Cas9
nCas9: Nicking Cas9
PAM: Protospacer adjacent motif
sgRNA: Single guide RNA
DNA: Deoxyribonucleic acid
RNA: Ribonucleic acid
CBE: Cytosine base editor
CDA: Cytidine deaminase
UGI: Uracil glycosylase inhibitor
OD_600_: Optical density at 600 nm
CDS: Coding sequence
sfGFP: Superfolder green fluorescent protein
5-FOA: 5-fluoroorotic acid
RM: Retriction-modification
REase: Restriction enzyme
MTase: Methyltransferase
CFU: Colony forming unit
