# Supplementary material for: Development of a CRISPR-based cytosine base editor for restriction-modification system inactivation to enhance transformation efficiency in Vibrio Sp. dhg
Source: J Biol Eng. 2025 Apr 9;19:30. doi: 10.1186/s13036-025-00500-4 (PMC11984283; doi:10.1186/s13036-025-00500-4)
Supplement: Supplementary file 1 — Supplementary Material 1 [file 13036_2025_500_MOESM1_ESM.docx]

**Supplementary information for**

Development of a CRISPR-based cytosine base editor for
restriction-modification system inactivation to enhance
transformation efficiency in *Vibrio* sp. dhg

Yang Jun Shon^a,1^, Dongyeop Baek^a,1^, Su Bin Jin^c^, Woo Jae Kim^a^,
Gyoo Yeol Jung^a,b,*^, Hyun Gyu Lim^c,*^

^a^Department of Chemical Engineering, Pohang University of Science and Technology, 77 Cheongam-Ro, Nam-Gu, Pohang, Gyeongbuk 37673, Korea

^b^School of Interdisciplinary Bioscience and Bioengineering, Pohang University of Science and Technology, 77 Cheongam-Ro, Nam-Gu, Pohang, Gyeongbuk 37673, Korea

^c^Department of Biological Sciences and Bioengineering, Inha University, 100 Inha-Ro, Michuhol-Gu, Incheon 22212, Korea

^1^ These authors contributed equally

^*^Corresponding authors: Gyoo Yeol Jung and Hyun Gyu Lim

Department of Chemical Engineering, Pohang University of Science and Technology, 77 Cheongam-Ro, Nam-Gu, Pohang, Gyeongbuk 37673, Korea

Tel.: +82-54-279-2391, Fax: +82-54-279-5528, E-mail: gyjung@postech.ac.kr

Department of Biological Sciences and Bioengineering, Inha University, 100 Inha-Ro, Michuhol-Gu, Incheon 22212, Korea

Tel.: +82-32-860-7538, Fax:+82-32-860-7726, E-mail: hyungyu.lim@inha.ac.kr

##

# Supplementary Tables

## Table S1. Strains used in this study

| **Strain** | **Description^a,b^** | **Source** |
| --- | --- | --- |
| *E. coli* Mach1-T1^®^ | Cloning host | Invitrogen |
| *Vibrio* sp. dhg | A novel alginate-metabolizing microorganism | [(Lim et al. 2019)](https://sciwheel.com/work/citation?ids=7145833&pre=&suf=&sa=0) |
| VDHG100 | *Vibrio* sp. dhg Δ*dns* | [(Lim et al. 2019)](https://sciwheel.com/work/citation?ids=7145833&pre=&suf=&sa=0) |
| VDHG *rpoB*^S531F^ | *Vibrio* sp. dhg Δ*dns rpoB*^S531F^ | This study |
| VDHG *pyrF*^W83*^ | *Vibrio* sp. dhg Δ*dns* Δ*pyrF^W83*^* | This study |
| RE1 | *Vibrio* sp. dhg Δ*dns* ΔRE1^a^ | This study |
| RE2 | *Vibrio* sp. dhg Δ*dns* ΔRE2^b^ | This study |
| RE3 | *Vibrio* sp. dhg Δ*dns* ΔRE1^a^ ΔRE2^b^ | This study |

^a^ indicates inactivation by stop codon formation in four REase genes of RE1 listed in Table 1
^b^ indicates inactivation by stop codon formation in three REase genes of RE2 listed in Table 1

##

## Table S2. Plasmids used in this study

| **Plasmid** | **Description^a^** | **Source** |
| --- | --- | --- |
| pACYCDuet™-1 | Source of pACYC vector (p15a ori-Cm^R^), and used for transformation assay as empty plasmid | Novagen |
| pJUMP24-1A | Source of pRO1600/ColE1 vector (pRO1600/ColE1 ori-Kan^R^) | [(Martínez-García et al. 2023)](https://sciwheel.com/work/citation?ids=14550576&pre=&suf=&sa=0) |
| pScI_dCas9-CDA-UL | Source of dCas9-CDA-UGIL | [(Banno et al. 2018)](https://sciwheel.com/work/citation?ids=5932550&pre=&suf=&sa=0) |
| pST_116_LVL2 cam | Source of sfGFP dropout system | [(Stukenberg et al. 2022)](https://sciwheel.com/work/citation?ids=15434337&pre=&suf=&sa=0) |
| pCBE1 | pACYC_P_BAD_-dCas9-CDAL, *araC* | This study |
| pCBE2 | pACYC_P_BAD_-dCas9-CDA-UGIL, *araC* | This study |
| psgRNA_sfGFP | pRO1600/ColE1_P_J23119_-sgRNA-sfGFP | This study |
| psgRNA_*rpoB* | pRO1600/ColE1_P_J23119_-sgRNA-*rpoB*^S531F^ | This study |
| psgRNA_*pyrF* | pRO1600/ColE1_P_J23119_-sgRNA-pyrF^W83*^ | This study |
| psgRNA_sgfp d/o_0-1 | pRO1600/ColE1_P_J23119_-sfGFP d/o  Level 0-1 (sfGFP dropout system for RE0-1) | This study |
| psgRNA_sgfp d/o_0-2 | pRO1600/ColE1_P_J23119_-sfGFP d/o  Level 0-2 (sfGFP dropout system for RE0-2) | This study |
| psgRNA_sgfp d/o_0-3 | pRO1600/ColE1_P_J23119_-sfGFP d/o  Level 0-3 (sfGFP dropout system for RE0-3) | This study |
| psgRNA_sgfp d/o_0-4 | pRO1600/ColE1_P_J23119_-sfGFP d/o  Level 0-4 (sfGFP dropout system for RE0-4) | This study |
| psgRNA_sgfp d/o_0-5 | pRO1600/ColE1_P_J23119_-sfGFP d/o  Level 0-5 (sfGFP dropout system for RE0-5) | This study |
| psgRNA_sgfp d/o_0-6 | pRO1600/ColE1_P_J23119_-sfGFP d/o  Level 0-6 (sfGFP dropout system for RE0-6) | This study |
| psgRNA_sgfp d/o_0-7 | pRO1600/ColE1_P_J23119_-sfGFP d/o  Level 0-7 (sfGFP dropout system for RE0-7) | This study |
| psgRNA_sgfp d/o_1 | pRO1600/ColE1_P_J23119_-sfGFP d/o  Level 1-1 (sfGFP dropout system for RE1) | This study |
| psgRNA_sgfp d/o_2 | pRO1600/ColE1_P_J23119_-sfGFP d/o  Level 1-2 (sfGFP dropout system for RE2) | This study |
| psgRNA_RE0-1 | pRO1600/ColE1_P_J23119_-sgRNA-RE0-1^a^ | This study |
| psgRNA_RE0-2 | pRO1600/ColE1_P_J23119_-sgRNA-RE0-2^a^ | This study |
| psgRNA_RE0-3 | pRO1600/ColE1_P_J23119_-sgRNA-RE0-3^a^ | This study |
| psgRNA_RE0-4 | pRO1600/ColE1_P_J23119_-sgRNA-RE0-4^a^ | This study |
| psgRNA_RE0-5 | pRO1600/ColE1_P_J23119_-sgRNA-RE0-5^a^ | This study |
| psgRNA_RE0-6 | pRO1600/ColE1_P_J23119_-sgRNA-RE0-6^a^ | This study |
| psgRNA_RE0-7 | pRO1600/ColE1_P_J23119_-sgRNA-RE0-7^a^ | This study |
| pMultisgRNA_RE1 | pRO1600/ColE1_P_J23119_-sgRNA-RE0-1^a^,  P_J23119_-sgRNA-RE0-2^a^,  P_J23119_-sgRNA-RE0-3^a^,  P_J23119_-sgRNA-RE0-4^a^ | This study |
| pMultisgRNA_RE2 | pRO1600/ColE1_P_J23119_-sgRNA-RE0-5^a^,  P_J23119_-sgRNA-RE0-6^a^,  P_J23119_-sgRNA-RE0-7^a^ | This study |

**^a^REase targets listed in Table 1**

## Table S3. Primers used in this study

| **Name** | **Sequence (5’ – 3’)^a,b^** |
| --- | --- |
| **CBE, sgRNA dual plasmid system cloning** | |
| pACYC_FWD | CTGCTGCCACCGCTGAGCAATAAC |
| UL-pACYC_REV | GTTATTGCTCAGCGGTGGCAGCAGTTATGCAACCAGTCCTAGCATCTTGATC |
| pACYC-Pbad_REV | CCATTTGACAGGCACATTATGCATCGAGCGCAACGCAATTAATGTAAGTTAGCTCAC |
| Pbad_FWD | TCGATGCATAATGTGCCTGTCAAATGG |
| Pbad-UTR_REV | TTCTCCCTCCGATGAACCAAACGTTTATTGACAAAAAAACGGGTATGGAGAAACAGTAGAGAG |
| UTR-dCas9_FWD | TCAATAAACGTTTGGTTCATCGGAGGGAGAAATGGATAAGAAATACTCAATAGGCTTAGCTATCGG |
| LVA-pACYC_F | GGACTGGTTGCATAACTGCTGC |
| CDA_R | AACAGCAGGACTCTTAGTGGTGTGG |
| pRO1600/ColE1_FWD | CTTCTGACTGAGTTGCACGCTG |
| pRO1600/ColE1_REV | CTCCGGTCTCACTCCAGAGAC |
| sgRNA_Ins_F | TCGACTCGTCTCTGGAGTGAGTTGACAGCTAGCTCAGTCCTAGGTATAATG |
| sgRNA_Ins_R | TCGACTCGTCTCACATTCACATTTCCCCGAAAAGTGCCAC |
| **Multiplex sgRNA plasmid cloning** | |
| gRNA-sgfp(d/o)_Ins_F | TAGACTTAGCAGAGACCGAAAGTGAAACGTGATTTCATG |
| gRNA-sgfp(d/o)_Ins_R | TCCAGCATAGCTCTGAAACTGAGACC |
| gRNA-sgfp(d/o)_Vec_F | TATACTGGTCTCAGTTTTAGAGCTAGAAATAGCAAGTTAAAATAAGGCTAG |
| gRNA-sgfp(d/o)_Vec_R | GACAATGGTCTCTGCTAGCATTATACCTAGGACTGAGCTAG |
| sgRNA_Lv0-1_Vec_GGF | GTACACTACTAGAGACGGCTGCAGGGAGTTGTCTTCGAAG |
| sgRNA_Lv0-1_Ins_GGR | TCGACTCGTCTCTAGTACACATTTCCCCGAAAAGTGCCAC |
| sgRNA_Lv0-2_Vec_GGF | GTACACAGGTAGAGACGGCTGCAGGGAGTTGTCTTCGAAG |
| sgRNA_Lv0-2_Vec_GGR | CTATGAAGTAAGAGACGGAATTCAGCGAAAAGCAGGTGTTCC |
| sgRNA_Lv0-2_Ins_GGF | TCGACTCGTCTCTACCTCACATTTCCCCGAAAAGTGCCAC |
| sgRNA_Lv0-2_Ins_GGR | TCGACTCGTCTCGTACTTGAGTTGACAGCTAGCTCAGTCCTAGG |
| sgRNA_Lv0-3_Vec_GGF | GTACACGCCTAGAGACGGCTGCAGGGAGTTGTCTTCGAAG |
| sgRNA_Lv0-3_Ins_GGR | TCGACTCGTCTCTAGGCCACATTTCCCCGAAAAGTGCCAC |
| sgRNA_Lv0-3_Vec_GGR | CTATGAACCTAGAGACGGAATTCAGCGAAAAGCAGGTGTTCC |
| sgRNA_Lv0-3_Ins_GGF | TCGACTCGTCTCGAGGTTGAGTTGACAGCTAGCTCAGTCCTAGG |
| sgRNA_Lv0-4_Vec_GGF | GTACACCGCTAGAGACGGCTGCAGGGAGTTGTCTTCGAAG |
| sgRNA_Lv0-4_Ins_GGR | TCGACTCGTCTCTAGCGCACATTTCCCCGAAAAGTGCCAC |
| sgRNA_Lv0-4_Vec_GGR | CTATGAAGGCAGAGACGGAATTCAGCGAAAAGCAGGTGTTCC |
| sgRNA_Lv0-4_Ins_GGF | TCGACTCGTCTCGGCCTTGAGTTGACAGCTAGCTCAGTCCTAGG |
| sgRNA_Lv0-5_Vec_GGR | CTATGAAGCGAGAGACGGAATTCAGCGAAAAGCAGGTGTTCC |
| sgRNA_Lv0-5_Ins_GGF | TCGACTCGTCTCGCGCTTGAGTTGACAGCTAGCTCAGTCCTAGG |
| sgRNA_Lv1-1_Vec_R | GTCAACTCACTCCAGAGACGCTCTAG |
| sgRNA_Lv1-1_Ins_F | GCTTCTAGAGCGTCTCTGGAGTGAG |
| sgRNA_Lv1-2_Ins_R | GCTACTAGTACGTCTCACATTCACATTTCC |
| Ptac-gam-bet_FWD | GAGTTTTGCAGGTGCCTTGGAACAC |
| pQE-R2 | CCAGATGGAGTTCTGAGGTCATTACTGG |
| MultigRNA_Vec_F | GTGGCACTTTTCGGGGAAATGTG |
| MultigRNA_Vec_R | ATTATACCTAGGACTGAGCTAGCTGTCAA |
| **gRNA exchange** |  |
| rpoB_gRNA_GF | ATTTCTGCATTGGGTCCTGGGTTTTAGAGCTAGAAATAGCAAGTTAAAATAAGGCTAG |
| rpoB_gRNA_GR | CCAGGACCCAATGCAGAAATGCTAGCATTATACCTAGGACTGAGCTAGC |
| pyrF_gRNA_GF | CATCCAAACACCCAGTTCCGGTTTTAGAGCTAGAAATAGCAAGTTAAAATAAGGCTAG |
| pyrF_gRNA_GR | CGGAACTGGGTGTTTGGATGGCTAGCATTATACCTAGGACTGAGCTAGC |
| sgfp_gRNA_F | **TAGC**AGCACTGCACGCCGTAGGTC |
| sgfp_gRNA_R | **AAAC**GACCTACGGCGTGCAGTGCT |
| RM0-1_gRNA_F | **TAGC**CCCATAGTTGCTGAGGTGTT |
| RM0-1_gRNA_R | **AAAC**AACACCTCAGCAACTATGGG |
| RM0-2_gRNA_F | **TAGC**ACCCAATTGAGGGAATTAAG |
| RM0-2_gRNA_R | **AAAC**CTTAATTCCCTCAATTGGGT |
| RM0-3_gRNA_F | **TAGC**ACGACAAGACCAAACTGCTG |
| RM0-3_gRNA_R | **AAAC**CAGCAGTTTGGTCTTGTCGT |
| RM0-4_gRNA_F | **TAGC**AGCCATCGTTTAGGGCTGAC |
| RM0-4_gRNA_R | **AAAC**GTCAGCCCTAAACGATGGCT |
| RM5_gRNA_F | **TAGC**CGTCAATGGCTAGTCGATAT |
| RM5_gRNA_R | **AAAC**ATATCGACTAGCCATTGACG |
| RM6_gRNA_F | **TAGC**ACAAGAGAGCTTGGATGTGG |
| RM6_gRNA_R | **AAAC**CCACATCCAAGCTCTCTTGT |
| RM7_gRNA_F | **TAGC**CCAAATTAGTAAAGAAGTAA |
| RM7_gRNA_R | **AAAC**TTACTTCTTTACTAATTTGG |
| **Mutagenesis site sequencing** | |
| rpoB_mut_FWD | GCCTAGGCGATCTTGATGCTGTTATG |
| rpoB_mut_REV | CACGTTTCGCAACTGCAGTTACAC |
| pyrF_mut_FWD | CTGATGCGCTTGCGTTTGTAGATC |
| pyrF_mut_REV | GTCACCTTGCTCAGAACCAGCAG |
| RM1_mut-check_FWD | GTACTGAGCTGGCTAATCGCATGG |
| RM1_mut-check_REV | GTTTCTTTCGGTGTCGCGGTTAGC |
| RM2_mut-check_FWD | GCTTATTGGGAGGAGTTATGGCTCG |
| RM2_mut-check_REV | GGTTAGACTGCTTGAACCCTCACTC |
| RM3_mut-check_FWD | CCAGCCACTCTGCTCATGCTG |
| RM3_mut-check_REV | CAGTGGCCGCAATCTGGTAGG |
| RM4_mut-check_FWD | GACGATTGGAGTGTAGCGACAGC |
| RM4_mut-check_REV | GCAGCAAGGTATTCCTCGCTACC |
| RM5_mut-check_FWD | GTTGACGGAGAGGACGCTTTAAATCG |
| RM5_mut-check_REV | CACGCCACGGCTTGATATGAGAAG |
| RM6_mut-check_FWD | GATTGGCAGTCGGCTAATAGAATTGTCG |
| RM6_mut-check_REV | GCTGATAATCCCAGAGCGGTTTATCG |
| RM7_mut-check_FWD | GGTCGCTGGGTTGATAATGTCTTTATCG |
| RM7_mut-check_REV | GGTCTTACTGATACGTCTCCGGACAC |

^a^ Overhangs used in PCR for Gibson and Golden Gate Assembly are underlined
^b^ Sticky ends of gRNA oligonucleotides are shown in bold

## Table S4. gRNA targeting genes in *Vibrio* sp. dhg

| **Target** | **Spacer sequence (5’ – 3’)** |
| --- | --- |
| *rpoB* | ATTTCTGCATTGGGTCCTGG |
| *pyrF* | CATCCAAACACCCAGTTCCG |
| RE0-1 | CCCATAGTTGCTGAGGTGTT |
| RE0-2 | ACCCAATTGAGGGAATTAAG |
| RE0-3 | ACGACAAGACCAAACTGCTG |
| RE0-4 | AGCCATCGTTTAGGGCTGAC |
| RE0-5 | CGTCAATGGCTAGTCGATAT |
| RE0-6 | ACAAGAGAGCTTGGATGTGG |
| RE0-7 | CCAAATTAGTAAAGAAGTAA |

##

# Supplementary Figures

## Figure S1. Rifampicin-resistance gain by CBE-mediated mutagenesis of *rpoB*


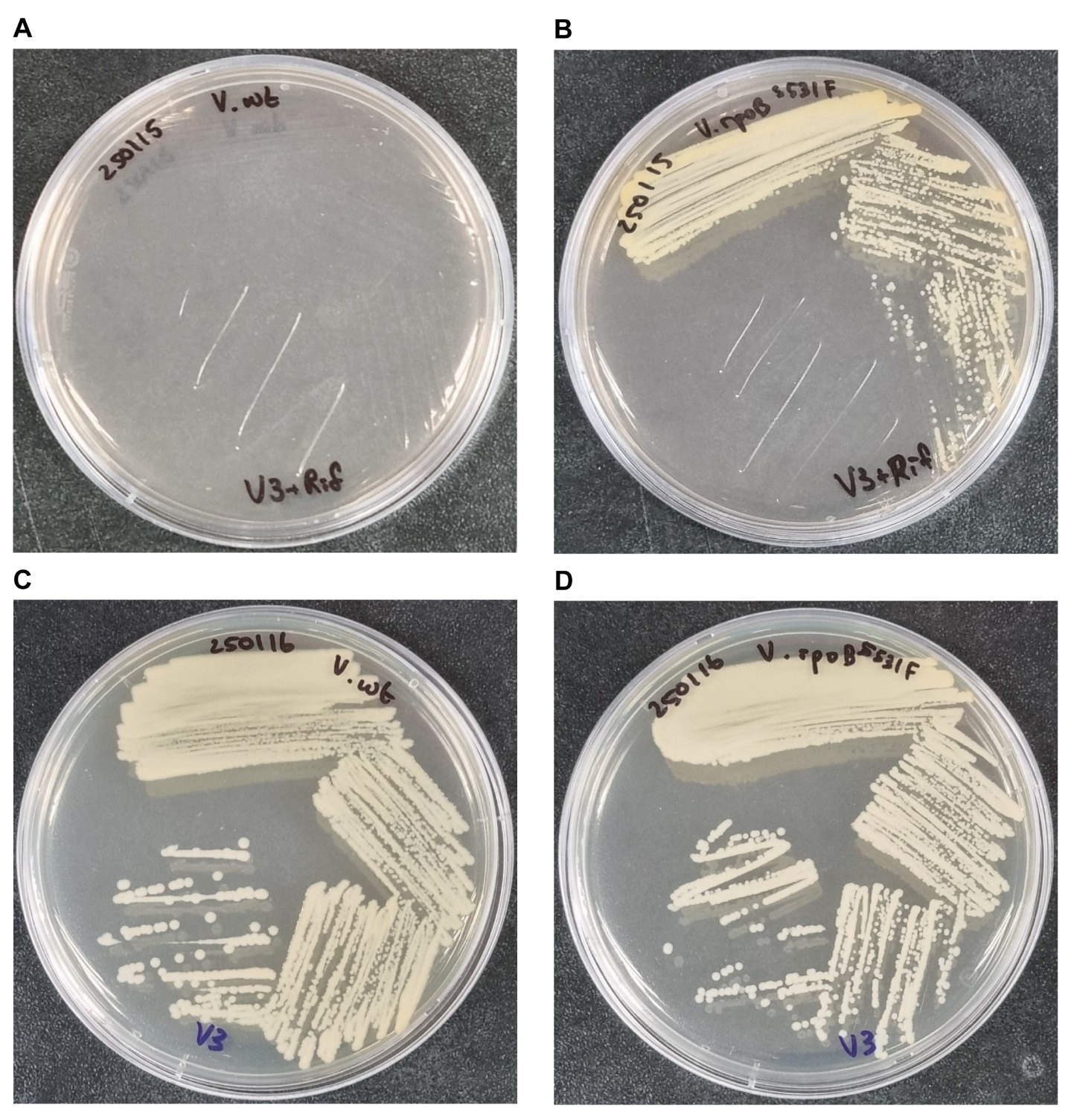


Streaking of (**A**) the wildtype strain, (**B**) *Vibrio* sp. dhg *rpoB*^S531F^ mutant strain on solid medium with rifampicin. Streaking of (**C**) the wildtype strain and (**D**) *Vibrio* sp. dhg *rpoB*^S531F^ mutant strain on nonselective medium shown as a positive control.

## Figure S2. 5-FOA-resistance increase by CBE-mediated mutagenesis of *pyrF*


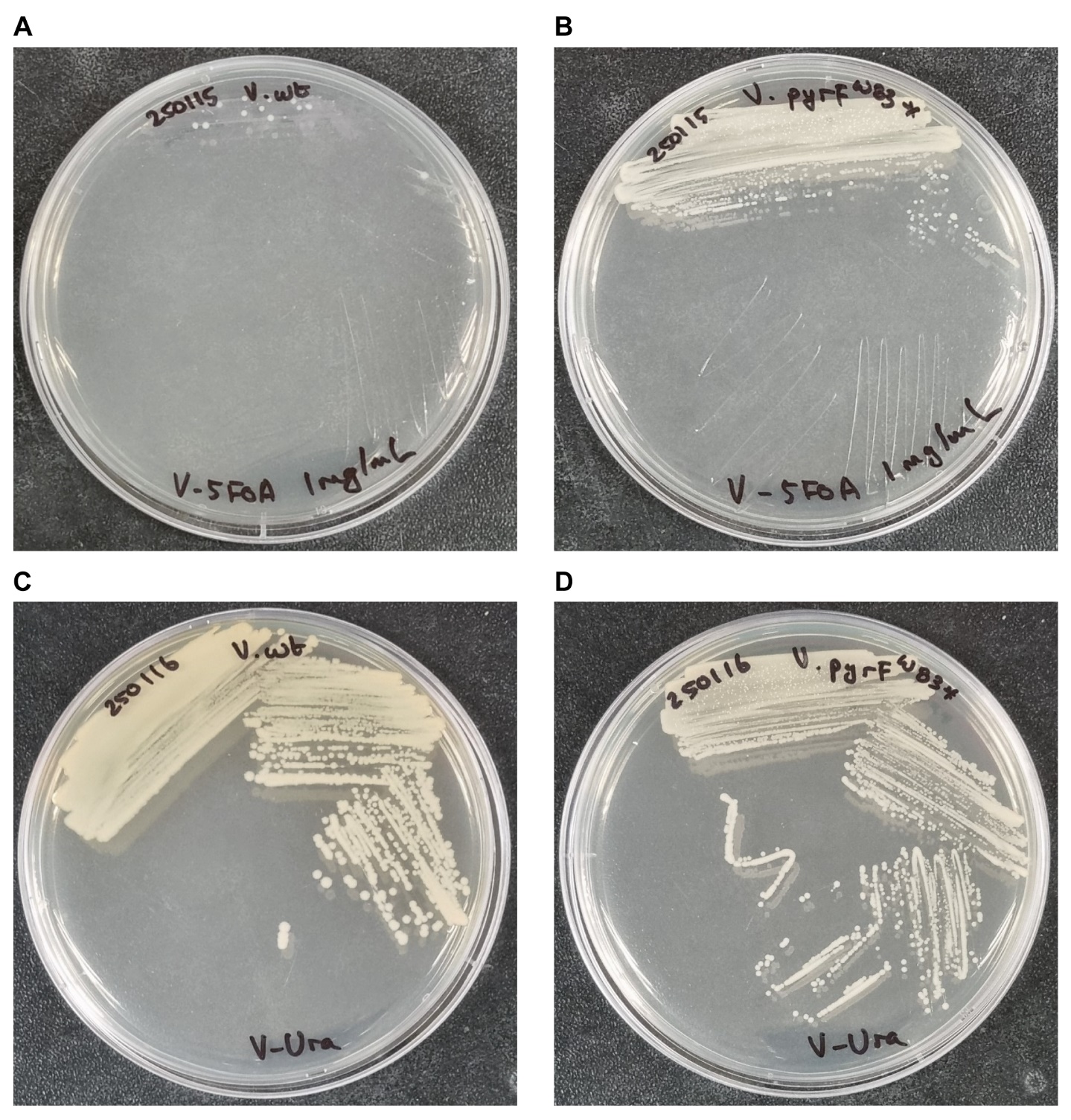


Streaking of (**A**) the wildtype strain, (**B**) *Vibrio* sp. dhg *pyrF*^W83*^ mutant strain on solid medium with 5-FOA and uracil. Streaking of (**C**) the wildtype and (**D**) *Vibrio* sp. dhg *pyrF*^W83*^ mutant strain on nonselective medium with uracil shown as a positive control.

## Figure S3. Multiplex base editing efficiency by target site


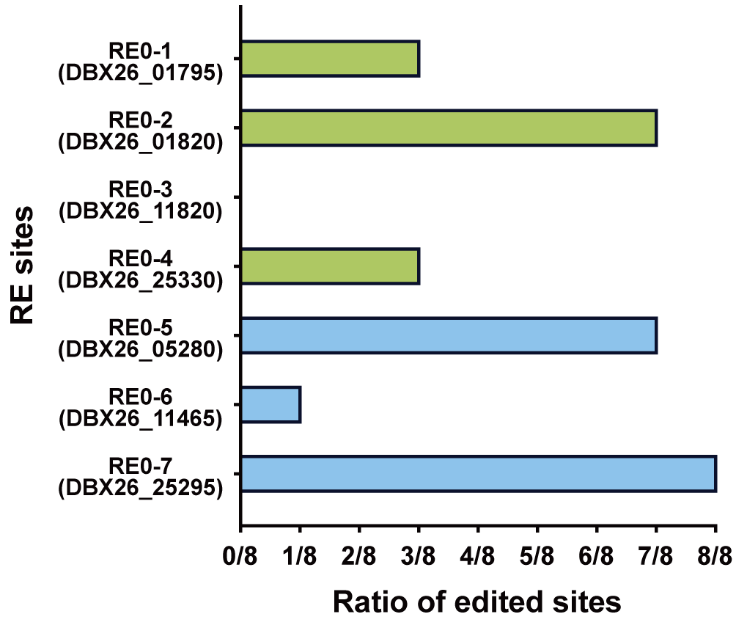


Multiplex base editing efficiency of RE1 targets (green) and RE2 targets (blue) regarding the ratio of edited colonies in each target site.
